# The COSMAM TRIAL a prospective cohort study of quality of life and cosmetic outcome in patients undergoing breast conserving surgery

**DOI:** 10.1186/s12885-018-4368-8

**Published:** 2018-04-23

**Authors:** Coriene J. L. M. Catsman, Martinus A. Beek, Adri C. Voogd, Paul G. H. Mulder, Ernest J. T. Luiten

**Affiliations:** 1 Department of Surgery, Amphia Hospital, Molengracht 21, 4818 CK Breda, The Netherlands; 2 Department of Epidemiology, Maastricht University, PO Box 616, 6200 MD Maastricht, The Netherlands; 3 Department of Research, Netherlands Comprehensive Cancer Organization (IKNL), Utrecht, The Netherlands

**Keywords:** Breast conserving surgery, Oncoplastic surgery, Breast cancer, Quality of life, Cosmetic result, Patient reported outcomes

## Abstract

**Background:**

Cosmetic result in breast cancer surgery is gaining increased interest. Currently, some 30–40% of the patients treated with breast conserving surgery (BCS) are dissatisfied with their final cosmetic result. In order to prevent disturbing breast deformity oncoplastic surgical techniques have been introduced. The extent of different levels of oncoplastic surgery incorporated in breast conserving surgery and its value with regard to cosmetic outcome, patient satisfaction and quality of life remains to be defined. The aim of this prospective cohort study is to investigate quality of life and satisfaction with cosmetic result in patients with breast cancer, undergoing standard lumpectomy versus level I or II oncoplastic breast conserving surgery.

**Methods:**

Female breast cancer patients scheduled for BCS, from 18 years of age, referred to our outpatient clinic from July 2015 are asked to participate in this study. General, oncologic and treatment information will be collected. Patient satisfaction will be scored preceding surgery, and at 1 month and 1 year follow up. Photographs of the breast will be used to score cosmetic result both by the patient, an independent expert panel and BCCT.Core software. Quality of life will be measured by using the BREAST-Q BCT, EORTC-QLQ and EQ-5D-5 L questionnaires.

**Discussion:**

The purpose of this prospective study is to determine the clinical value of different levels of oncoplastic techniques in breast conserving surgery, with regard to quality of life and cosmetic result. Analysis will be carried out by objective measurements of the final cosmetic result in comparison with standard breast conserving surgery. The results of this study will be used to development of a clinical decision model to guide the use oncoplastic surgery in future BCS.

**Trial registration:**

Central Commission of Human Research (CCMO), The Netherlands: NL54888.015.15.

Medical Ethical Commission (METC), Maxima Medical Centre, Veldhoven, The Netherlands: 15.107.

Dutch Trial Register: NTR5665, retrospectively registered, 02-25-2016.

**Electronic supplementary material:**

The online version of this article (10.1186/s12885-018-4368-8) contains supplementary material, which is available to authorized users.

## Background

Breast conserving surgery (BCS) is carried out since the late seventies of the former century. Studies with more than 20 years of follow up have shown its equivalency to mastectomy with regard to loco regional control and overall survival [1–4]. Currently, the majority of woman with breast cancer (50–70%) is treated with BCS [5]. Apart from oncological adequate resection achievement of a good cosmetic result and high patient satisfaction are essential components of successful BCS. Psychological morbidity due to disfigurement following surgery for breast cancer therapy in patients were first reported in the early fifties [6]. However more thorough studies on QoL (psychosocial, physical and sexual well-being) originate only form the last 2 decades. This growing awareness for preservation of body image, cosmetic result and QoL is attenuated by the increasing prevalence of patients who survive breast cancer [7].

Today, some 30–40% of the patients report that they are disappointed with the final cosmetic result of BCS [8]. Breast asymmetry after BCS is significantly correlated with poor psychosocial functioning and fear of recurrence, which has negative impact on QoL [8, 9]. Poor cosmetic result is caused by contour defects, asymmetry of the breasts, malposition of the nipple, adhesions, and retractions due to large resection volumes, which can be attenuated by subsequent radiation therapy [10]. Tumor to breast ratio as well as localization of the tumour is accepted factors with regard to the risk to obtain an unsatisfactory cosmetic result. Several studies have reported that cosmetic failure rates are significantly higher when the tumour is located in the inner half of the breast, if lumpectomy volume exceeds > 20% of the breast or more than 50–85 ml regardless of the size of the breast [11–14].

Both Audretsch and Gabka first introduced oncoplastic breast conserving surgery in the early nineties [15–17]. In order to prevent breast deformity many different oncoplastic techniques have been proposed to reshape and/or modify the appearance of the breast. Clough et al. described several of these oncoplastic surgical techniques in a quadrant-per-quadrant atlas [18]. Oncoplastic surgery (OPS) incorporated in BCS involves reconstruction of the resection defects by volume displacement techniques using adjacent breast tissue. With regard to volume displacement techniques 2 levels of OPS are recognized, based on the extent of the resection and the corresponding mammoplasty techniques [18]. Level I OPS includes local re-arrangement of volume defects of < 10%, and level II OPS includes the volume displacement technique for volume defects of more than 10–30% volume.

Recognizing the risk of a potentially unfavourable cosmetic outcome is the first step in deciding when to incorporate OPS in BCS. Application of OPS is reported to expand the possibility of BCS thereby still achieving acceptable or good cosmetic results and QoL [19]. On the contrary, in a small subgroup of a recent Dutch retrospective study in 125 patients (74% BCS and 26% OPS) it was suggested that patients in whom conventional BCS was carried out scored significantly better than those treated with OPS when scored by BCCT.Core cosmetic result scoring software [20].

The exact role as well as the extent of OPS in BCS in order to ameliorate cosmetic outcome, patient satisfaction and quality of life remains rather ill defined. The former is partly due to lack of high quality studies on this issue.

In order to identify factors that affect cosmetic outcome and influence QoL a standardized method to evaluate cosmetic result is warranted. Currently, different methods are used to evaluate the cosmetic result of BCS, including panel evaluation using a photo scoring method, computer programs that objectively and automatically evaluate breast aesthetics by using patient photographs (BCCT.Core), the breast retraction assessment to measure breast asymmetry and patient opinion using QoL forms [21–24].

Patient satisfaction with cosmetic result, preservation of body image and QoL is mostly assessed by QoL questionnaires that mainly originate from plastic surgery reduction techniques that are not specifically validated for breast cancer patients undergoing BCS [25]. The BREAST-Q questionnaire is a validated survey instrument especially developed for patients undergoing breast surgery, measuring QoL (sexual, mental and physical wellbeing and satisfaction with breast). Recently the BREAST-Q BCT module was developed, which is specifically designed for patients undergoing BCS [26].

Proper evaluation of the possible additional value of OPS incorporated in standard BCS is limited due to lack of uniformity in measurements, validated quality of life questionnaires, objective scoring of cosmetic results and patient satisfaction. Current studies also lack a robust level of evidence partly due to study design, small numbers of patients, incomplete follow up and incomplete data on patient reported outcomes, including evaluations in which patients score photographs of their own breasts. In the first Dutch guideline of OPS an urgent need for studies comparing all objective and subjective scoring systems prospectively in well-designed study protocols is recommended [27].

## Methods

The aim of this prospective cohort study is to assess the additional value of different levels of (oncoplastic) BCS in breast cancer patients with, with regard to quality of life and objective cosmetic outcome, in comparison with conventional lumpectomy combined simple closure of the cavity. Secondly, this study will examine the value of different quality of life scoring systems in evaluating satisfaction with cosmetic results in patients undergoing BCS, with or without the use of oncoplastic techniques.

### Participants: Inclusion and exclusion criteria

All female patients referred to our outpatient clinic from June 2015, eligible for BCS and BCS with OPS that are older than 18 years, will be evaluated for inclusion in this cohort study. Patients will be included in the study directly after signing informed consent and before the operation. Informed consent will be obtained and explained by a qualified nurse practitioner in the outpatient clinic. Patients who are not familiar with Dutch language or with a history of breast cancer and/or radiation therapy in the head/neck/axillary or breast region in the past will be excluded.

### Data collection

For all patients in the study data will be collected prior to surgery (T0), 4 weeks after surgery (T1) and 1 year after surgery (T2) (Table 1: Data Collection). These data include:

**Table 1:** Data collection

	Pre-operative (*T* = 0)	1 month post-operative (*T* = 1)	1 year post-operative (*T* = 2)
General questionnaire	+	+	+
Breast-Q Pre-operative	+		
Breast-Q Post-operative		+	+
EORTC-QLQ and EQ-5D-5 L	+	+	+
Breast Photo	+	+	+
Diagnostics & Treatment	–	–	–
Breast Photo Scoring Patient	+	+	+
Breast Photo Scoring BCCT.Core/Expert panel	–	–	–

- = Professional + = Patient

### Patient characteristics

- Oncologic diagnostics and treatment characteristics
- Patient questionnaires
- Photographs of the breast

#### Patient characteristics

Prior to surgery (*T* = 0) and 4 weeks after surgery (*T* = 1) patients will be asked to fill out a general questionnaire regarding marital status, ethnicity, breast size, body mass index (BMI), educational level and work. One year after surgery (*T* = 2) patients will be asked to complete a final general questionnaire including questions concerning the use treatments for lymphedema, return to normal daily activities and work and their opinion of the final cosmetic result.

#### Diagnostics and treatment characteristics

Baseline characteristics including oncologic data will be collected of all patients containing information about histology, volume of the breast, hormone receptor status, tumour location and tumour size on magnetic resonance imaging (MRI), amount of removed breast tissue during lumpectomy, axillary surgery, re-excision, adjuvant radiotherapy and hormone therapy.

#### Patient questionnaires

Preoperatively (*T* = 0), patients will be asked to fill out the pre-operative breast-Q for BCT questionnaire (Additional file 1) measuring QoL, which contains questions related to psychosocial, physical and sexual wellbeing. Both at *T* = 1 and *T* = 2, patients will be asked to fill out the post-operative breast-Q for BCT questionnaire, which also contains questions about psychosocial, physical and sexual wellbeing. The breast-Q is a patient reported outcome instrument designed to standardize evaluation outcomes [26]. At *T* = 0, *T* = 1, T = 2 patients will also be asked to fill out the EQ-5D-5L (Additional file 2) and the EORTC-QLQ (Additional files 3 and 4) and the EQ-5D-5 L. EQ-5D-5 L is a health related QoL questionnaire (mobility, self-care, usual activities, pain/discomfort, anxiety and depression) in which patients rate their own health [28]. The EORTC-QLQ breast module is a questionnaire developed to assess the QoL of cancer patients [29].

#### Photographs of the breast

Standardized photographs of the breast of all patients will be made prior to surgery (*T* = 0), 4 weeks after surgery (*T* = 1) and 1 year after surgery (*T* = 2) by a professional medical photographer*.*

#### Cosmetic result

The value of different scoring systems to evaluate satisfaction with the cosmetic outcome in patients undergoing conventional lumpectomy and versus BCS and OPS will be examined [30]. Photographs of the breast will be assessed with quantitative and qualitative scoring systems. Qualitative scoring will be carried out by using a standardized score list for cosmetic result. Both an expert panel and patients will be asked to score the result. Since strong variation between observers is common when measuring cosmetic outcome, the expert panel will consist of at least five members, including both professionals and non-professionals, both male and female with different backgrounds [24]. The panel members will score all photographs individually at all three time points. The pre-operative cosmetic appearance of the breast (*T* = 0) will be used as a baseline and will be scored by a qualitative scoring system by using the adjusted version of the patient self-assessment scoring system, developed by Vos et al. The original 11 items patient self-assessment scoring system of Vos et al., will be used to score cosmetic result at *T* = 1 and *T* = 2 [23].

Haloua et al. have suggested that patient self-evaluation of the cosmetic outcome should be incorporated in future studies apart from other evaluation methods. Self-evaluation reflects the psychological adaptation of the patient to the appearance of the breast [30]. All patients will be asked to score their own photographs at all three time points. Cosmetic appearance of the breast at *T* = 0 and will be used as baseline. A patient version of the scoring list of *Vos* et al. to score the photographs will be used at *T* = 1 and *T* = 2. Evaluation of all scoring systems will be based on the percentage of the total score.

**Fig. 1:**
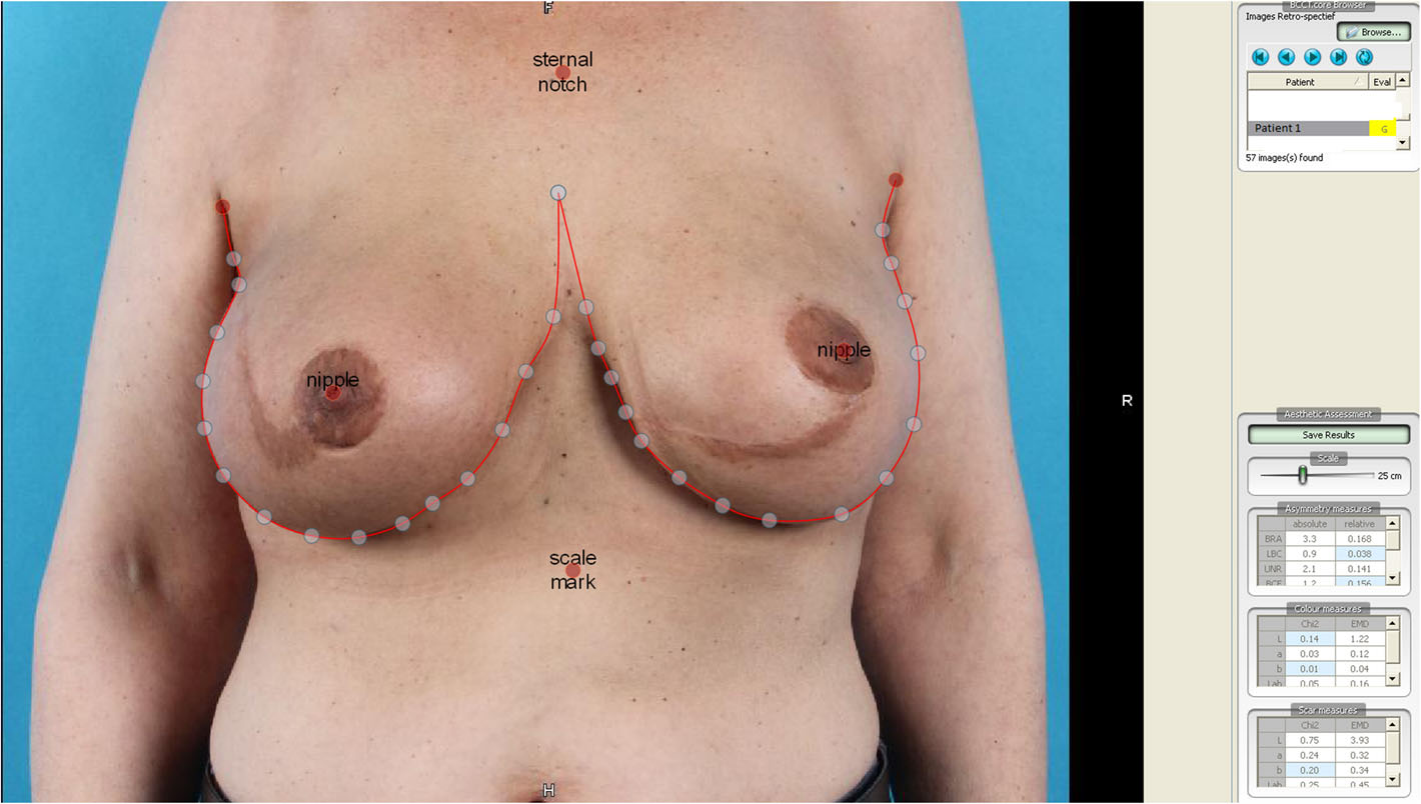
Example of BCCT.core software

Quantitative scoring will be conducted by using BCCT.Core software that automatically evaluates breast aesthetics. BCCT.Core software is an objective method for the evaluation of cosmetic result in BCS [31]. BCCT.Core software was proven to provide valid cosmetic outcome scores compared to a panel evaluation and is used in several studies [30]. The investigator digitally marks the nipples, axillae and sternum, jugular notch; BCCT.Core software then identifies the relative difference between both breasts and nipples and the relative difference between nipples and inframammary fold difference. (Figure 1: example of BCCT.Core software).

### Objectives

The aim of this single-centre prospective registration cohort study is to assess the additional value of different levels of oncoplastic BCS in patients with breast, with regard to quality of life and cosmetic outcome by objective measurements of the final cosmetic result in comparison with patients undergoing conventional lumpectomy. Secondly, study trial will examine the value of different quality of life scoring systems in evaluating satisfaction with cosmetic results in patients undergoing BCS.

### Treatment allocation

All patients undergoing breast conserving surgery are requested to participate in this study irrespective of the pre-planned surgical technique to be performed. The decision with regard to the type of surgical technique is not strictly defined but depends on the preference of the individual surgeon taking into account tumour location, breast/tumour ratio, the quality of the breast tissue, breast size and degree of ptosis. In general, surgeons are recommended to perform oncoplastic surgery in case of expected resections of more than 10% of the breast volume as well as large lesions in the upper medial and lower medial and lower lateral quadrants. Planning is done by using shared decision-making. Including informing patients that more extensive procedures may need larger incisions. Surgical procedures are divided as follows:

1. Standard lumpectomy with/without minor volume replacement:
2. Level 1 oncoplastic surgery:
3. Level 2 oncoplastic surgery:

### Withdrawal and replacement

Patients can leave the study at any time for any reason. The investigator can decide to withdraw a subject from the study for urgent medical reasons. Patients will not be replaced after withdrawal and will have regular outpatient clinic check-up. These patients will be considered as “lost to follow-up”.

### Sample size

Annually, approximately 450 new women are diagnosed with breast cancer in our surgical outpatient clinic (2011). Approximately 75% of these women are initially treated with BCS. Three operation types are considered: standard, level I and level II. The cosmetic score at 4 weeks is considered the primary outcome variable. From the presently available data the pre-operation level of the cosmetic score appeared to be comparable between the three operation types (*n* = 151; *p* = 0.60). The power calculation will be based on the upper limit of the 95% confidence interval of the pooled within operation type residual standard deviation of the cosmetic score at 4 weeks, adjusted for its pre-operation level. Based on estimates obtained from the present data this residual SD will be set at 16 points on a (0–100)-scale. Two comparisons between operation types will be made: level I with standard and level II with standard. According to Cohen’s *d* a medium effect of 0.5 SD (= 8 points) will be considered to be minimally detectable. The test size alpha of either comparison with the standard operation will be set at a two-sided level of 0.027 according to Dunnett’s *t*, resulting in an overall alpha of 0.05 (two-sided). The resulting sample size per operation type is 75 (80% power) or 100 (90% power). Purpose is to recruit at least 75 patients per operation type, including the patients recruited so far.

### Statistical analysis

Raw data summaries of patient, tumour and treatment characteristics will be made by operation type. Spearman’s correlation and Pearson’s correlation will be used in a descriptive way to assess the correlation of the mean score of total expert panel score, total patient score and automated calculations of the BCCT.Core software. Cosmetic result and quality of life will be measured at *T* = 0, *T* = 1 and *T* = 2. Difference in (changes from baseline of) satisfaction of cosmetic results will be compared between patients undergoing BCS without OPS with those with OPS using linear mixed modelling, with adjustment made for the baseline (*T* = 0) measurement of cosmetic scoring and for other potentially confounding factors such as age, location, size of the tumour and re-operation. The quality of life instruments (EQ5D5L, BREAST-Q, EORTC QLQ C30 and EORTC QLQ BR23) will be analysed similarly after using the prescribed scoring methods when appropriate. The partial correlation of the level of the cosmetic results with the baseline level of each of the quality of life instruments will be analysed after adjustment for operation type. At *T* = 1 and *T* = 2 the partial correlation will be analysed after adjustment for operation type and the baseline level of both the cosmetic result and the quality of life instrument at hand, so as to assess the correlation between changes at *T* = 1 and *T* = 2 from what is known at *T* = 0. Effect of resection volume on the cosmetic score at *T* = 1 and *T* = 2 will be analysed using linear mixed modelling with adjustment for the potentially confounding effects of baseline cosmetic score, age, education, body mass index, tumour quadrant, operation type, possible re-operation and/or complications.

All statistical analysis will be performed with SPSS version 23.0 from IBM Software.

### Adverse events

No adverse events as a result of this study are expected, as patients do not undergo any additional interventions. If any adverse events of one of these surgical methods are identified they will be reported to the medical ethical commission and hospital board.

### Protocol changes

Important protocol modifications will be first submitted to a medical ethical commission and the hospital board. In case of approval all participants and the Dutch trial register will be informed.

## Discussion

The COSMAM Trial is a first prospective cohort study that aims to define the role of different levels of additional OPS in BCS for breast cancer patient. This study compares objective and subjective scoring systems prospectively and is approved by a Dutch medical ethical board.

Since randomization for this type of surgery is virtually impossible the study observes the results of daily practice. This will give a real world experience of current breast saving treatment in an oncological breast cancer centre with multiple surgeons performing different levels of OPS in different patients according to their personal preference. We do realize that in the ideal setting patients with similar tumour types in the same quadrant of the breast should preferably be randomized between lumpectomy with oncoplastic surgery and conventional lumpectomy. However from both an ethical and practical point of view we feel that carrying out such a study could possibly affect cosmetic result and quality of life. Especially in patients with an expected removal of > 10% of the total breast volume as well as large lesions in the upper medial and lower medial and lower lateral quadrants. Based on the reports that application of OPS expands the possibility of BCS thereby still achieving acceptable or good cosmetic results and QoL [19]. Without OPS in these patients a mastectomy would be considered because of high chance of deformation of the breast performing a standard lumpectomy.

All patients who are scheduled for BCS are asked to participate in this study. Our study population is considered to be representative a reflection of the Dutch community.

Despite literature describing the use of OPS there is no clear international standard describing the indications for application of different levels of OPS neither which technique is indicated in relation to the tumour site and the density of the breast tissue. Based on current literature OPS is recommended in patients with a tumour situated in the inner half of the breast, when lumpectomy exceeds > 20% of total breast volume or more than 50–85 ml, regardless of the size of the breast [11–14, 27]. The techniques that are currently used and to what extent they are carried out mainly depends on the surgeon’s preference and expertise.

To implement OPS as a standard additional technique in BCS more data are needed regarding the clinical value of which patients benefit from OPS, thereby not only taking into account tumour characteristics and location but also type of breast tissue and opinion of the patient. The aim of the COSMAM trial is to assess the additional value of different levels of oncoplastic BCS, with regard to QoL and cosmetic outcome by objective measurements of the final cosmetic result in comparison with patients undergoing conventional BCS. The patient and tumour characteristics correlated with a satisfying cosmetic result and QoL after OPS may give valuable information for development of decision-making tree when to use OPS and which technique will give the most satisfying and oncologic safe result.

The implementation of a decision-making tree for the indication of OPS follows the trend of adjusting breast cancer treatment to tumour type and location as well as specific patient characteristics. By personalizing BCS we will not only focus on the medical treatment of breast cancer patients but also on individual specific needs and personal preferences of the patient as a human being.

## Conclusion

The purpose of this prospective study is to assess the clinical value of addition of different oncoplastic techniques in breast conserving surgery, with regard to quality of life and cosmetic outcome by objective measurements of the final cosmetic result in comparison with standard breast conserving surgery. The results of this study can be used for development of a clinical decision model to guide the use of oncoplastic breast techniques in order to establish personalized oncologic safe and cosmetic satisfying breast conserving treatment.

## Additional files

Additional file 1: Sample Breast Q – BCT Module, Quality of Life Questionnaire. (PDF 335 kb)

Additional file 2: Sample EQ5D5L, Quality of Life Questionnaire. (PDF 106 kb)

Additional file 3: Sample EORTC QLQ C30, Quality of Life Questionnaire. (PDF 886 kb)

Additional file 4: Sample EORTC QLQ B23, Quality of life Questionnaire. (PDF 225 kb)

## Abbreviations

BCS: Breast Conserving Surgery
BMI: Body Mass Index (length^2^/weight)
ER: Estrogen Receptor
Her2Neu: Human Epidermal Growth Factor Receptor 2
LR: Local recurrence
LRFS: Local recurrence free survival
MRI: Magnetic Resonance Imaging
OPS: Oncoplastic Breast Conversing Surgery
pBAD: Relative breast area difference
pBCE: Relative breast compliance evaluation
pBRA: Relative breast retraction assessment
PR: Progesterone receptor
pUNR: Relative upward nipple retraction
QoL: Quality of life
SN: Sentinel node procedure
SPSS: Statistical package for the social sciences
