# Supplementary material for: The COSMAM TRIAL a prospective cohort study of quality of life and cosmetic outcome in patients undergoing breast conserving surgery
Source: BMC Cancer. 2018 Apr 23;18:456. doi: 10.1186/s12885-018-4368-8 (PMC5914027; doi:10.1186/s12885-018-4368-8)
Supplement: Supplementary file 1 — Sample Breast Q – BCT Module, Quality of Life Questionnaire. (PDF 335 kb) [file 12885_2018_4368_MOESM1_ESM.pdf]

BREAST-Q™ - BREAST CONSERVING THERAPY MODULE (PREOPERATIVE) VERSION 2.0
SATISFACTION WITH BREASTS

With your breast area in mind, in the past week, how satisfied or dissatisfied have you been with:

	Very Dissatisfied	Somewhat Dissatisfied	Somewhat Satisfied	Very Satisfied
a. How you look in the mirror clothed?	1	2	3	4
b. How comfortably your bras fit?	1	2	3	4
c. Being able to wear clothing that is more fitted?	1	2	3	4
d. How you look in the mirror unclothed?	1	2	3	4

BREAST-Q® VERSION 2.0 © Memorial Sloan Kettering Cancer Center and The University of British Columbia, 2017, All rights reserved

---

Note to Investigators: This scale can be used independently of the other scales. This scale is exactly the same across the three Breast Cancer Preoperative Modules (i.e. Mastectomy, Reconstruction, and Breast Conserving Therapy).

The BREAST-Q, authored by Drs. Andrea Pusic, Anne Klassen and Stefan Cano, is the copyright of Memorial Sloan Kettering Cancer Center and The University of British Columbia (Copyright ©2017, Memorial Sloan Kettering Cancer Center and the University of British Columbia). The BREAST-Q has been provided under license from Memorial Sloan Kettering Cancer Center and must not be copied, distributed or used in any way without the prior consent of Memorial Sloan Kettering Cancer Center.

Review Copy
Do not use without permission

BREAST-Q™ - BREAST CONSERVING THERAPY MODULE (PREOPERATIVE) VERSION 2.0
SATISFACTION WITH BREASTS CONVERSION TABLE

Instructions: If missing data is less than 50% of the scale's items, insert the mean of the completed items. Use the Conversion Table below to convert the raw scale summed score into a score from 0 (worst) to 100 (best). Higher scores reflect a better outcome.

SUM SCORE	EQUIVALENT RASCH TRANSFORMED SCORE (0-100)
4	0
5	23
6	29
7	34
8	39
9	44
10	48
11	53
12	58
13	64
14	71
15	82
16	100

Review Copy
Do not use without permission

BREAST-Q™ - BREAST CONSERVING THERAPY MODULE (PREOPERATIVE) VERSION 2.0
PSYCHOSOCIAL WELL-BEING

With your breast area in mind, in the past week, how often have you felt:

	None of the time	A little of the time	Some of the time	Most of the time	All of the time
a. Confident in a social setting?	1	2	3	4	5
b. Emotionally able to do the things that you want to do?	1	2	3	4	5
c. Emotionally healthy?	1	2	3	4	5
d. Of equal worth to other women?	1	2	3	4	5
e. Self-confident?	1	2	3	4	5
f. Feminine in your clothes?	1	2	3	4	5
g. Accepting of your body?	1	2	3	4	5
h. Normal?	1	2	3	4	5
i. Like other women?	1	2	3	4	5
j. Attractive?	1	2	3	4	5

BREAST-Q® VERSION 2.0 © Memorial Sloan Kettering Cancer Center and The University of British Columbia, 2017, All rights reserved

Review Copy
Do not use without permission

Note to Investigators: This scale can be used independently of the other scales. This scale is exactly the same across the three Breast Cancer Preoperative and Postoperative Modules (i.e. Mastectomy, Reconstruction, and Breast Conserving Therapy).

The BREAST-Q, authored by Drs. Andrea Pusic, Anne Klassen and Stefan Cano, is the copyright of Memorial Sloan Kettering Cancer Center and The University of British Columbia (Copyright ©2017, Memorial Sloan Kettering Cancer Center and the University of British Columbia). The BREAST-Q has been provided under license from Memorial Sloan Kettering Cancer Center and must not be copied, distributed or used in any way without the prior consent of Memorial Sloan Kettering Cancer Center.

BREAST-Q™ - BREAST CONSERVING THERAPY MODULE (PREOPERATIVE) VERSION 2.0 PSYCHOSOCIAL WELL-BEING CONVERSION TABLE

Instructions: If missing data is less than 50% of the scale's items, insert the mean of the completed items. Use the Conversion Table below to convert the raw scale summed score into a score from 0 (worst) to 100 (best). Higher scores reflect a better outcome.

SUM SCORE	EQUIVALENT RASCH TRANSFORMED SCORE (0-100)
10	0
11	13
12	18
13	21
14	24
15	27
16	29
17	31
18	32
19	34
20	35
21	37
22	38
23	39
24	41
25	42
26	43
27	44
28	45
29	47
30	48
31	49
32	50
33	52
34	53
35	55
36	56
37	58
38	60
39	62
40	64
41	66
42	69
43	71
44	74
45	77
46	80
47	83
48	87
49	93
50	100

Review Copy
Do not use without permission

BREAST-Q™ - BREAST CONSERVING THERAPY MODULE (PREOPERATIVE) VERSION 2.0
PHYSICAL WELL-BEING: CHEST

In the past week, how often have you experienced:

	None of the time	Some of the time	All of the time
a. Pain in the muscles of your chest?	1	2	3
b. Difficulty lifting or moving your arms?	1	2	3
c. Difficulty sleeping because of discomfort in your breast area?	1	2	3
d. Tightness in your breast area?	1	2	3
e. Pulling in your breast area?	1	2	3
f. Nagging feeling in your breast area?	1	2	3
g. Tenderness in your breast area?	1	2	3
h. Sharp pains in your breast area?	1	2	3
i. Aching feeling in your breast area?	1	2	3
j. Throbbing feeling in your breast area?	1	2	3

BREAST-Q® VERSION 2.0 © Memorial Sloan Kettering Cancer Center and The University of British Columbia, 2017, All rights reserved

Review Copy
Do not use without permission

Note to Investigators: This scale can be used independently of the other scales. This scale is exactly the same across the three Breast Cancer Preoperative Modules (i.e. Mastectomy, Reconstruction, and Breast Conserving Therapy).

The BREAST-Q, authored by Drs. Andrea Pusic, Anne Klassen and Stefan Cano, is the copyright of Memorial Sloan Kettering Cancer Center and The University of British Columbia (Copyright ©2017, Memorial Sloan Kettering Cancer Center and the University of British Columbia). The BREAST-Q has been provided under license from Memorial Sloan Kettering Cancer Center and must not be copied, distributed or used in any way without the prior consent of Memorial Sloan Kettering Cancer Center.

BREAST-Q™ - BREAST CONSERVING THERAPY MODULE (PREOPERATIVE) VERSION 2.0
PHYSICAL WELL-BEING: CHEST CONVERSION TABLE

Instructions: Recode items a, b, c, d, e, f, g, h, i, and j as follows: "None of the time" = 3; "Some of the time" = 2; "All of the time" = 1. If missing data is less than 50% of the scale's items, insert the mean of the completed items. Use the Conversion Table below to convert the raw scale summed score into a score from 0 (worst) to 100 (best). Higher scores reflect a better outcome.

SUM SCORE	EQUIVALENT RASCH TRANSFORMED SCORE (0-100)
10	0
11	8
12	14
13	20
14	24
15	28
16	32
17	36
18	40
19	45
20	50
21	55
22	60
23	64
24	68
25	72
26	76
27	80
28	85
29	92
30	100

Review Copy
Do not use without permission

BREAST-Q™ - BREAST CONSERVING THERAPY MODULE (PREOPERATIVE) VERSION 2.0
SEXUAL WELL-BEING

Thinking of your sexuality, how often do you generally feel:

	None of the time	A little of the time	Some of the time	Most of the time	All of the time
a. Sexually attractive in your clothes?	1	2	3	4	5
b. Comfortable/at ease during sexual activity?	1	2	3	4	5
c. Confident sexually?	1	2	3	4	5
d. Satisfied with your sex-life?	1	2	3	4	5
e. Confident sexually about how your breast area looks when unclothed?	1	2	3	4	5
f. Sexually attractive when unclothed?	1	2	3	4	5

BREAST-Q® VERSION 2.0 © Memorial Sloan Kettering Cancer Center and The University of British Columbia, 2017, All rights reserved

Note to Investigators: This scale can be used independently of the other scales. This scale is exactly the same across the three Breast Cancer Preoperative and Postoperative Modules (i.e. Mastectomy, Reconstruction, and Breast Conserving Therapy). The following statement can be added to the stem to provide an opportunity for the patient to decline completing this scale. 'The following questions ask about your sexual well-being. If you are uncomfortable answering these questions or do not feel that they apply to you, please check the box and skip the questions that follow.'

The BREAST-Q, authored by Drs. Andrea Pusic, Anne Klassen and Stefan Cano, is the copyright of Memorial Sloan Kettering Cancer Center and The University of British Columbia (Copyright ©2017, Memorial Sloan Kettering Cancer Center and the University of British Columbia). The BREAST-Q has been provided under license from Memorial Sloan Kettering Cancer Center and must not be copied, distributed or used in any way without the prior consent of Memorial Sloan Kettering Cancer Center.

Review Copy
Do not use without permission

BREAST-Q™ - BREAST CONSERVING THERAPY MODULE (PREOPERATIVE) VERSION 2.0
SEXUAL WELL-BEING CONVERSION TABLE

Instructions: If missing data is less than 50% of the scale's items, insert the mean of the completed items. Use the Conversion Table below to convert the raw scale summed score into a score from 0 (worst) to 100 (best). Higher scores reflect a better outcome.

SUM SCORE	EQUIVALENT RASCH TRANSFORMED SCORE (0-100)
6	0
7	14
8	20
9	24
10	27
11	31
12	34
13	36
14	39
15	41
16	43
17	46
18	48
19	50
20	53
21	56
22	59
23	62
24	66
25	70
26	74
27	79
28	84
29	91
30	100

Review Copy
Do not use without permission

BREAST-Q^{TM} - BREAST CONSERVING THERAPY MODULE (POSTOPERATIVE) VERSION 2.0
SATISFACTION WITH BREASTS

The following questions are about your breasts and your breast cancer treatment (by treatment, we mean lumpectomy with or without radiation). If you have had a lumpectomy and radiation of both breasts, answer these questions thinking of the breast you are least satisfied with.
With your breasts in mind, in the past week, how satisfied or dissatisfied have you been with:

	Very Dissatisfied	Somewhat Dissatisfied	Somewhat Satisfied	Very Satisfied
a. How you look in the mirror clothed?	1	2	3	4
b. The shape of your lumpectomy breast when you are wearing a bra?	1	2	3	4
c. How normal you feel in your clothes?	1	2	3	4
d. Being able to wear clothing that is more fitted?	1	2	3	4
e. How your lumpectomy breast sits/hangs?	1	2	3	4
f. How smoothly shaped your lumpectomy breast looks?	1	2	3	4
g. The contour (outline) of your lumpectomy breast?	1	2	3	4
h. How equal in size your breasts are to each other?	1	2	3	4
i. How normal your lumpectomy breast looks?	1	2	3	4
j. How much your breasts look the same?	1	2	3	4
k. How you look in the mirror unclothed?	1	2	3	4

BREAST-Q® VERSION 2.0 © Memorial Sloan Kettering Cancer Center and The University of British Columbia, 2017, All rights reserved

Review Copy
Do not use without permission

Note to Investigators: This scale can be used independently of the other scales.

The BREAST-Q, authored by Drs. Andrea Pusic, Anne Klassen and Stefan Cano, is the copyright of Memorial Sloan Kettering Cancer Center and The University of British Columbia (Copyright ©2017, Memorial Sloan Kettering Cancer Center and the University of British Columbia). The BREAST-Q has been provided under license from Memorial Sloan Kettering Cancer Center and must not be copied, distributed or used in any way without the prior consent of Memorial Sloan Kettering Cancer Center.

BREAST-Q^{TM} - BREAST CONSERVING THERAPY MODULE (POSTOPERATIVE) VERSION 2.0
SATISFACTION WITH BREASTS CONVERSION TABLE

Instructions: If missing data is less than 50% of the scale's items, insert the mean of the completed items. Use the Conversion Table below to convert the raw scale summed score into a score from 0 (worst) to 100 (best). Higher scores reflect a better outcome.

SUM SCORE	EQUIVALENT RASCH TRANSFORMED SCORE (0-100)
**11**	**0**
**12**	**15**
**13**	**20**
**14**	**24**
**15**	**26**
**16**	**29**
**17**	**31**
**18**	**33**
**19**	**35**
**20**	**36**
**21**	**38**
**22**	**40**
**23**	**42**
**24**	**43**
**25**	**45**
**26**	**46**
**27**	**48**
**28**	**50**
**29**	**51**
**30**	**53**
**31**	**55**
**32**	**57**
**33**	**59**
**34**	**61**
**35**	**63**
**36**	**65**
**37**	**67**
**38**	**69**
**39**	**72**
**40**	**75**
**41**	**78**
**42**	**82**
**43**	**88**
**44**	**100**

Review Copy
Do not use without permission

BREAST-Q^{TM} - BREAST CONSERVING THERAPY MODULE (POSTOPERATIVE) VERSION 2.0
ADVERSE EFFECTS OF RADIATION

If you have had radiation on both breasts, answer these questions thinking of the breast you are least satisfied with. With your radiated breast(s) in mind, in the past week, how much have you been bothered by:

	Not at all	A little	A lot
a. Your radiated breast skin looking different (e.g. too dark or too light)?	1	2	3
b. Marks on your breast skin caused by radiation (e.g. small visible blood vessels)?	1	2	3
c. Your radiated breast skin feeling dry?	1	2	3
d. Your radiated breast skin feeling sore (sensitive) when touched (e.g. changes in water temperature when you bathe/shower)?	1	2	3
e. Your radiated breast skin feeling unnaturally thick (rough, tough) when you touch it?	1	2	3
f. Your radiated breast skin feeling irritated by clothing that you wear?	1	2	3

BREAST-Q® VERSION 2.0 © Memorial Sloan Kettering Cancer Center and The University of British Columbia, 2017, All rights reserved

Review Copy Do not use without permission

Note to Investigators: This scale can be used independently of the other scales and should only be completed by patients who have had radiation. The following statement can be added to the stem to provide an opportunity for the patient to decline completing this scale. 'If you have not had radiation, please check the box and skip the questions that follow.' This scale is exactly the same across the three Breast Cancer Postoperative Modules (i.e. Mastectomy, Reconstruction, and Breast Conserving Therapy).

The BREAST-Q, authored by Drs. Andrea Pusic, Anne Klassen and Stefan Cano, is the copyright of Memorial Sloan Kettering Cancer Center and The University of British Columbia (Copyright ©2017, Memorial Sloan Kettering Cancer Center and the University of British Columbia). The BREAST-Q has been provided under license from Memorial Sloan Kettering Cancer Center and must not be copied, distributed or used in any way without the prior consent of Memorial Sloan Kettering Cancer Center.

BREAST-Q^{TM} - BREAST CONSERVING THERAPY MODULE (POSTOPERATIVE) VERSION 2.0
ADVERSE EFFECTS OF RADIATION CONVERSION TABLE

Instructions: Rescore items a, b, c, d, e and f as follows: “Not at all” = 3; “A little” = 2; “A lot” = 1.If missing data is less than 50% of the scale’s items, insert the mean of the completed items. Use the Conversion Table below to convert the raw scale summed score into a score from 0 (worst) to 100 (best). Higher scores reflect a better outcome.

SUM SCORE	EQUIVALENT RASCH TRANSFORMED SCORE (0-100)
**6**	0
**7**	11
**8**	21
**9**	29
**10**	36
**11**	44
**12**	51
**13**	58
**14**	65
**15**	71
**16**	78
**17**	87
**18**	100

Review Copy
Do not use without permission

BREAST-Q™ - BREAST CONSERVING THERAPY MODULE (POSTOPERATIVE) VERSION 2.0
PSYCHOSOCIAL WELL-BEING

With your breasts in mind, in the past week, how often have you felt:

	None of the time	A little of the time	Some of the time	Most of the time	All of the time
a. Confident in a social setting?	1	2	3	4	5
b. Emotionally able to do the things that you want to do?	1	2	3	4	5
c. Emotionally healthy?	1	2	3	4	5
d. Of equal worth to other women?	1	2	3	4	5
e. Self-confident?	1	2	3	4	5
f. Feminine in your clothes?	1	2	3	4	5
g. Accepting of your body?	1	2	3	4	5
h. Normal?	1	2	3	4	5
i. Like other women?	1	2	3	4	5
j. Attractive?	1	2	3	4	5

BREAST-Q® VERSION 2.0 © Memorial Sloan Kettering Cancer Center and The University of British Columbia, 2017, All rights reserved

Review Copy
Do not use without permission

Note to Investigators: This scale can be used independently of the other scales. This scale is exactly the same across the three Breast Cancer Preoperative and Postoperative Modules (i.e. Mastectomy, Reconstruction, and Breast Conserving Therapy).

The BREAST-Q, authored by Drs. Andrea Pusic, Anne Klassen and Stefan Cano, is the copyright of Memorial Sloan Kettering Cancer Center and The University of British Columbia (Copyright ©2017, Memorial Sloan Kettering Cancer Center and the University of British Columbia). The BREAST-Q has been provided under license from Memorial Sloan Kettering Cancer Center and must not be copied, distributed or used in any way without the prior consent of Memorial Sloan Kettering Cancer Center.

BREAST-Q^{TM} - BREAST CONSERVING THERAPY MODULE (POSTOPERATIVE) VERSION 2.0
PSYCHOSOCIAL WELL-BEING CONVERSION TABLE

Instructions: If missing data is less than 50% of the scale's items, insert the mean of the completed items. Use the Conversion Table below to convert the raw scale summed score into a score from 0 (worst) to 100 (best). Higher scores reflect a better outcome.

SUM SCORE	EQUIVALENT RASCH TRANSFORMED SCORE (0-100)
10	0
11	13
12	18
13	21
14	24
15	27
16	29
17	31
18	32
19	34
20	35
21	37
22	38
23	39
24	41
25	42
26	43
27	44
28	45
29	47
30	48
31	49
32	50
33	52
34	53
35	55
36	56
37	58
38	60
39	62
40	64
41	66
42	69
43	71
44	74
45	77
46	80
47	83
48	87
49	93
50	100

Review Copy
Do not use without permission

BREAST-Q™ - BREAST CONSERVING THERAPY MODULE (POSTOPERATIVE) VERSION 2.0
PHYSICAL WELL-BEING: CHEST

In the past week, how often have you experienced:

	None of the time	Some of the time	All of the time
a. Difficulty lifting or moving your arms?	1	2	3
b. Difficulty sleeping because of discomfort in your breast area?	1	2	3
c. Tightness in your breast area?	1	2	3
d. Pulling in your breast area?	1	2	3
e. Tenderness in your breast area?	1	2	3
f. Sharp pains in your breast area?	1	2	3
g. Aching feeling in your breast area?	1	2	3
h. Difficulty laying on the side of your lumpectomy breast?	1	2	3
i. Swelling of the arm (lymphedema) on the side(s) that you had your breast surgery?	1	2	3

BREAST-Q® VERSION 2.0 © Memorial Sloan Kettering Cancer Center and The University of British Columbia, 2017, All rights reserved

Note to Investigators: This scale can be used independently of the other scales.

The BREAST-Q, authored by Drs. Andrea Pusic, Anne Klassen and Stefan Cano, is the copyright of Memorial Sloan Kettering Cancer Center and The University of British Columbia (Copyright ©2017, Memorial Sloan Kettering Cancer Center and the University of British Columbia). The BREAST-Q has been provided under license from Memorial Sloan Kettering Cancer Center and must not be copied, distributed or used in any way without the prior consent of Memorial Sloan Kettering Cancer Center.

Review Copy
Do not use without permission

BREAST-Q™ - BREAST CONSERVING THERAPY MODULE (POSTOPERATIVE) VERSION 2.0
PHYSICAL WELL-BEING: CHEST CONVERSION TABLE

Instructions: Items 'h' and 'I' are stand-alone items that are not included in the scale score. Recode items a, b, c, d, e, f, and g as follows: "None of the time" = 3; "Some of the time" = 2; "All of the time" = 1. If missing data is less than 50% of the scale's items, insert the mean of the completed items. Use the Conversion Table below to convert the raw scale summed score into a score from 0 (worst) to 100 (best). Higher scores reflect a better outcome.

SUM SCORE	EQUIVALENT RASCH TRANSFORMED SCORE (0-100)
**7**	**0**
**8**	**13**
**9**	**21**
**10**	**27**
**11**	**33**
**12**	**38**
**13**	**45**
**14**	**52**
**15**	**60**
**16**	**66**
**17**	**71**
**18**	**76**
**19**	**82**
**20**	**89**
**21**	**100**

Review Copy
Do not use without permission

BREAST-Q^{TM} - BREAST CONSERVING THERAPY MODULE (POSTOPERATIVE) VERSION 2.0
SEXUAL WELL-BEING

Thinking of your sexuality, since your lumpectomy surgery, how often do you generally feel:

	None of the time	A little of the time	Some of the time	Most of the time	All of the time
a. Sexually attractive in your clothes?	1	2	3	4	5
b. Comfortable/at ease during sexual activity?	1	2	3	4	5
c. Confident sexually?	1	2	3	4	5
d. Satisfied with your sex-life?	1	2	3	4	5
e. Confident sexually about how your breast(s) look when unclothed?	1	2	3	4	5
f. Sexually attractive when unclothed?	1	2	3	4	5

BREAST-Q® VERSION 2.0 © Memorial Sloan Kettering Cancer Center and The University of British Columbia, 2017, All rights reserved

Review Copy Do not use without permission

Note to Investigators: This scale can be used independently of the other scales. This scale is exactly the same across the three Breast Cancer Preoperative and Postoperative Modules (i.e. Mastectomy, Reconstruction, and Breast Conserving Therapy).The following statement can be added to the stem to provide an opportunity for the patient to decline completing this scale. 'The following questions ask about your sexual well-being. If you are uncomfortable answering these questions or do not feel that they apply to you, please check the box and skip the questions that follow.'

The BREAST-Q, authored by Drs. Andrea Pusic, Anne Klassen and Stefan Cano, is the copyright of Memorial Sloan Kettering Cancer Center and The University of British Columbia (Copyright ©2017, Memorial Sloan Kettering Cancer Center and the University of British Columbia). The BREAST-Q has been provided under license from Memorial Sloan Kettering Cancer Center and must not be copied, distributed or used in any way without the prior consent of Memorial Sloan Kettering Cancer Center.

BREAST-Q^{TM} - BREAST CONSERVING THERAPY MODULE (POSTOPERATIVE) VERSION 2.0
SEXUAL WELL-BEING CONVERSION TABLE

Review Copy
Do not use without permission

Instructions: If missing data is less than 50% of the scale's items, insert the mean of the completed items. Use the Conversion Table below to convert the raw scale summed score into a score from 0 (worst) to 100 (best). Higher scores reflect a better outcome.

SUM SCORE	EQUIVALENT RASCH TRANSFORMED SCORE (0-100)
6	0
7	14
8	20
9	24
10	27
11	31
12	34
13	36
14	39
15	41
16	43
17	46
18	48
19	50
20	53
21	56
22	59
23	62
24	66
25	70
26	74
27	79
28	84
29	91
30	100

BREAST-Q™ - BREAST CONSERVING THERAPY MODULE (POSTOPERATIVE) VERSION 2.0
PATIENT EXPERIENCE: SATISFACTION WITH INFORMATION

How satisfied or dissatisfied were you with the information you received from your breast surgeon about:

	Very Dissatisfied	Somewhat Dissatisfied	Somewhat Satisfied	Very Satisfied
a. The possible need for radiation depending on the surgery you have (mastectomy vs lumpectomy)?	1	2	3	4
b. The options you were given regarding types of breast cancer surgery (mastectomy vs lumpectomy)?	1	2	3	4
c. How your survival would be the same with either type of surgery (mastectomy vs lumpectomy)?	1	2	3	4
d. Healing and recovery time?	1	2	3	4
e. What your treatment plan would involve if the cancer is found in your lymph nodes?	1	2	3	4
f. How much pain to expect during recovery?	1	2	3	4
g. Possible complications?	1	2	3	4
h. How the chances that the cancer would come back would be the same with either type of surgery (mastectomy vs lumpectomy)?	1	2	3	4
i. What you could expect your lumpectomy breast to look like after surgery?	1	2	3	4
j. What the lumpectomy scars would look like?	1	2	3	4
k. What size you could expect your breast to be after lumpectomy surgery?	1	2	3	4
l. What shape you could expect your breast to be after lumpectomy surgery?	1	2	3	4

BREAST-Q® VERSION 2.0 © Memorial Sloan Kettering Cancer Center and The University of British Columbia, 2017, All rights reserved

Preview Copy
Do not use without permission

Note to Investigators: This scale can be used independently of the other scales. Depending on the use of this scale, you may wish to add the following statement to the stem for clarity. 'These questions ask about the surgeon who performed your most recent surgery.'

The BREAST-Q, authored by Drs. Andrea Pusic, Anne Klassen and Stefan Cano, is the copyright of Memorial Sloan Kettering Cancer Center and The University of British Columbia (Copyright ©2017, Memorial Sloan Kettering Cancer Center and the University of British Columbia). The BREAST-Q has been provided under license from Memorial Sloan Kettering Cancer Center and must not be copied, distributed or used in any way without the prior consent of Memorial Sloan Kettering Cancer Center.

BREAST-Q™ - BREAST CONSERVING THERAPY MODULE (POSTOPERATIVE) VERSION 2.0
PATIENT EXPERIENCE: SATISFACTION WITH INFORMATION CONVERSION TABLE

Instructions: If missing data is less than 50% of the scale's items, insert the mean of the completed items. Use the Conversion Table below to convert the raw scale summed score into a score from 0 (worst) to 100 (best). Higher scores reflect a better outcome.

SUM SCORE	EQUIVALENT RASCH TRANSFORMED SCORE (0-100)
12	0
13	8
14	15
15	20
16	24
17	27
18	30
19	33
20	35
21	37
22	38
23	40
24	42
25	43
26	45
27	46
28	47
29	49
30	50
31	51
32	53
33	54
34	55
35	57
36	58
37	60
38	62
39	64
40	66
41	68
42	71
43	73
44	76
45	80
46	85
47	91
48	100

Review Copy
Do not use without permission

BREAST-Q™ - BREAST CONSERVING THERAPY MODULE (POSTOPERATIVE) VERSION 2.0
PATIENT EXPERIENCE: SATISFACTION WITH INFORMATION

How satisfied or dissatisfied were you with the information you received from your radiation oncologist about:

	Very Dissatisfied	Somewhat Dissatisfied	Somewhat Satisfied	Very Satisfied
a. How much time each radiation treatment session would take?	1	2	3	4
b. The position you would be in during the radiation (i.e. on your back or stomach)?	1	2	3	4
c. Why you would need radiation therapy after lumpectomy?	1	2	3	4
d. How much of your breast would be radiated?	1	2	3	4
e. How the radiation beam would feel?	1	2	3	4
f. How to care for your skin during the course of radiation treatment (moisturizing, washing)?	1	2	3	4
g. That you would end up with permanent skin markings (tattoos) on your breast?	1	2	3	4
h. How tired you might feel during your radiation treatment in general?	1	2	3	4
i. How the radiation might change your skin over time (color, texture)?	1	2	3	4
j. How the radiation might change how your breast looks over time?	1	2	3	4
k. Potential problems (late effects) that radiation might cause down the road?	1	2	3	4

BREAST-Q® VERSION 2.0 © Memorial Sloan Kettering Cancer Center and The University of British Columbia, 2017, All rights reserved

Review Copy
Do not use without permission

Note to Investigators: This scale can be used independently of the other scales. Depending on the use of this scale, you may wish to add the following statement to the stem for clarity. 'These questions ask about the oncologist who performed your most recent radiation treatment.'

The BREAST-Q, authored by Drs. Andrea Pusic, Anne Klassen and Stefan Cano, is the copyright of Memorial Sloan Kettering Cancer Center and The University of British Columbia (Copyright ©2017, Memorial Sloan Kettering Cancer Center and the University of British Columbia). The BREAST-Q has been provided under license from Memorial Sloan Kettering Cancer Center and must not be copied, distributed or used in any way without the prior consent of Memorial Sloan Kettering Cancer Center.

BREAST-Q™ - BREAST CONSERVING THERAPY MODULE (POSTOPERATIVE) VERSION 2.0
PATIENT EXPERIENCE: SATISFACTION WITH INFORMATION CONVERSION TABLE

Instructions: If missing data is less than 50% of the scale's items, insert the mean of the completed items. Use the Conversion Table below to convert the raw scale summed score into a score from 0 (worst) to 100 (best). Higher scores reflect a better outcome.

SUM SCORE	EQUIVALENT RASCH TRANSFORMED SCORE (0-100)
11	0
12	18
13	23
14	26
15	29
16	31
17	33
18	35
19	37
20	38
21	40
22	41
23	42
24	44
25	45
26	46
27	48
28	49
29	50
30	52
31	53
32	55
33	56
34	58
35	60
36	62
37	64
38	67
39	70
40	73
41	77
42	82
43	90
44	100

Review Copy
Do not use without permission

BREAST-Q™ - BREAST CONSERVING THERAPY MODULE (POSTOPERATIVE) VERSION 2.0
PATIENT EXPERIENCE: SATISFACTION WITH SURGEON

These questions ask about your breast surgeon. Did you feel that he/she:

	Definitely Disagree	Somewhat Disagree	Somewhat Agree	Definitely Agree
a. Was professional?	1	2	3	4
b. Gave you confidence?	1	2	3	4
c. Involved you in the decision-making process?	1	2	3	4
d. Was reassuring?	1	2	3	4
e. Answered all your questions?	1	2	3	4
f. Made you feel comfortable?	1	2	3	4
g. Was thorough?	1	2	3	4
h. Was easy to talk to?	1	2	3	4
i. Understood what you wanted?	1	2	3	4
j. Was sensitive?	1	2	3	4
k. Made time for your concerns?	1	2	3	4
l. Was available when you had concerns?	1	2	3	4

BREAST-Q® VERSION 2.0 © Memorial Sloan Kettering Cancer Center and The University of British Columbia, 2017, All rights reserved

Review Copy
Do not use without permission

Note to Investigators: This scale can be used independently of the other scales. This scale is exactly the same across all BREAST-Q Postoperative Modules. Depending on the use of this scale, you may wish to add the following statement to the stem for clarity. 'These questions ask about the surgeon who performed your most recent surgery.'

The BREAST-Q, authored by Drs. Andrea Pusic, Anne Klassen and Stefan Cano, is the copyright of Memorial Sloan Kettering Cancer Center and The University of British Columbia (Copyright ©2017, Memorial Sloan Kettering Cancer Center and the University of British Columbia). The BREAST-Q has been provided under license from Memorial Sloan Kettering Cancer Center and must not be copied, distributed or used in any way without the prior consent of Memorial Sloan Kettering Cancer Center.

BREAST-Q™ - BREAST CONSERVING THERAPY MODULE (POSTOPERATIVE) VERSION 2.0
PATIENT EXPERIENCE: SATISFACTION WITH SURGEON CONVERSION TABLE

Instructions: If missing data is less than 50% of the scale's items, insert the mean of the completed items. Use the Conversion Table below to convert the raw scale summed score into a score from 0 (worst) to 100 (best). Higher scores reflect a better outcome.

SUM SCORE	EQUIVALENT RASCH TRANSFORMED SCORE (0-100)
12	0
13	13
14	18
15	22
16	25
17	27
18	29
19	31
20	33
21	35
22	36
23	38
24	39
25	41
26	42
27	44
28	45
29	46
30	48
31	50
32	51
33	53
34	55
35	57
36	59
37	61
38	63
39	65
40	67
41	70
42	72
43	75
44	78
45	82
46	86
47	92
48	100

Review Copy
Do not use without permission

BREAST-Q™ - BREAST CONSERVING THERAPY MODULE (POSTOPERATIVE) VERSION 2.0
PATIENT EXPERIENCE: SATISFACTION WITH MEDICAL TEAM

These questions ask about members of the medical team other than the surgeon. Did you feel that they:

	Definitely Disagree	Somewhat Disagree	Somewhat Agree	Definitely Agree
a. Were professional?	1	2	3	4
b. Treated you with respect?	1	2	3	4
c. Were knowledgeable?	1	2	3	4
d. Were friendly and kind?	1	2	3	4
e. Made you feel comfortable?	1	2	3	4
f. Were thorough?	1	2	3	4
g. Made time for your concerns?	1	2	3	4

BREAST-Q® VERSION 2.0 © Memorial Sloan Kettering Cancer Center and The University of British Columbia, 2017, All rights reserved

Review Copy
Do not use without permission

Note to Investigators: This scale can be used independently of the other scales. This scale is exactly the same across all BREAST-Q Postoperative Modules. Depending on the use of this scale, you may modify the stem wording to fit your clinical environment. (e.g. medical team may include nurses, physician assistants, or other licensed independent practitioners)

The BREAST-Q, authored by Drs. Andrea Pusic, Anne Klassen and Stefan Cano, is the copyright of Memorial Sloan Kettering Cancer Center and The University of British Columbia (Copyright ©2017, Memorial Sloan Kettering Cancer Center and the University of British Columbia). The BREAST-Q has been provided under license from Memorial Sloan Kettering Cancer Center and must not be copied, distributed or used in any way without the prior consent of Memorial Sloan Kettering Cancer Center.

BREAST-Q[TM] - BREAST CONSERVING THERAPY MODULE (POSTOPERATIVE) VERSION 2.0
PATIENT EXPERIENCE: SATISFACTION WITH MEDICAL TEAM CONVERSION TABLE

Instructions: If missing data is less than 50% of the scale's items, insert the mean of the completed items. Use the Conversion Table below to convert the raw scale summed score into a score from 0 (worst) to 100 (best). Higher scores reflect a better outcome.

SUM SCORE	EQUIVALENT RASCH TRANSFORMED SCORE (0-100)
7	0
8	13
9	19
10	23
11	27
12	30
13	34
14	37
15	40
16	43
17	46
18	49
19	53
20	57
21	61
22	66
23	70
24	75
25	80
26	85
27	91
28	100

Review Copy
Do not use without permission

BREAST-Q™ - BREAST CONSERVING THERAPY MODULE (POSTOPERATIVE) VERSION 2.0
PATIENT EXPERIENCE: SATISFACTION WITH OFFICE STAFF

These questions ask about members of the office staff (e.g. secretaries). Did you feel that they:

	Definitely Disagree	Somewhat Disagree	Somewhat Agree	Definitely Agree
a. Were professional?	1	2	3	4
b. Treated you with respect?	1	2	3	4
c. Were knowledgeable?	1	2	3	4
d. Were friendly and kind?	1	2	3	4
e. Made you feel comfortable?	1	2	3	4
f. Were thorough?	1	2	3	4
g. Made time for your concerns?	1	2	3	4

BREAST-Q® VERSION 2.0 © Memorial Sloan Kettering Cancer Center and The University of British Columbia, 2017, All rights reserved

Review Copy
Do not use without permission

Note to Investigators: This scale can be used independently of the other scales. This scale is exactly the same across all BREAST-Q Postoperative Modules. Depending on the use of this scale, you may modify the stem wording to fit your office environment. (e.g. office or clinic nurse)

The BREAST-Q, authored by Drs. Andrea Pusic, Anne Klassen and Stefan Cano, is the copyright of Memorial Sloan Kettering Cancer Center and The University of British Columbia (Copyright ©2017, Memorial Sloan Kettering Cancer Center and the University of British Columbia). The BREAST-Q has been provided under license from Memorial Sloan Kettering Cancer Center and must not be copied, distributed or used in any way without the prior consent of Memorial Sloan Kettering Cancer Center.

BREAST-Q^{TM} - BREAST CONSERVING THERAPY MODULE (POSTOPERATIVE) VERSION 2.0
PATIENT EXPERIENCE: SATISFACTION WITH OFFICE STAFF CONVERSION TABLE

Instructions: If missing data is less than 50% of the scale's items, insert the mean of the completed items. Use the Conversion Table below to convert the raw scale summed score into a score from 0 (worst) to 100 (best). Higher scores reflect a better outcome.

SUM SCORE	EQUIVALENT RASCH TRANSFORMED SCORE (0-100)
7	0
8	11
9	17
10	21
11	25
12	28
13	32
14	36
15	39
16	42
17	46
18	49
19	53
20	57
21	63
22	68
23	73
24	77
25	82
26	87
27	93
28	100

Review Copy
Do not use without permission
