# Supplementary material for: The COSMAM TRIAL a prospective cohort study of quality of life and cosmetic outcome in patients undergoing breast conserving surgery
Source: BMC Cancer. 2018 Apr 23;18:456. doi: 10.1186/s12885-018-4368-8 (PMC5914027; doi:10.1186/s12885-018-4368-8)
Supplement: Supplementary file 4 — Sample EORTC QLQ B23, Quality of life Questionnaire. (PDF 225 kb) [file 12885_2018_4368_MOESM4_ESM.pdf]

EORTC QLQ - BR23

Patients sometimes report that they have the following symptoms or problems. Please indicate the extent to which you have experienced these symptoms or problems during the past week.

**During the past week:**	**Not at All**	**A Little**	**Quite a Bit**	**Very Much**
31. Did you have a dry mouth?	1	2	3	4
32. Did food and drink taste different than usual?	1	2	3	4
33. Were your eyes painful, irritated or watery?	1	2	3	4
34. Have you lost any hair?	1	2	3	4
35. Answer this question only if you had any hair loss: Were you upset by the loss of your hair?	1	2	3	4
36. Did you feel ill or unwell?	1	2	3	4
37. Did you have hot flushes?	1	2	3	4
38. Did you have headaches?	1	2	3	4
39. Have you felt physically less attractive as a result of your disease or treatment?	1	2	3	4
40. Have you been feeling less feminine as a result of your disease or treatment?	1	2	3	4
41. Did you find it difficult to look at yourself naked?	1	2	3	4
42. Have you been dissatisfied with your body?	1	2	3	4
43. Were you worried about your health in the future?	1	2	3	4

**During the past <u>four</u> weeks:**	**Not at All**	**A Little**	**Quite a Bit**	**Very Much**
44. To what extent were you interested in sex?	1	2	3	4
45. To what extent were you sexually active? (with or without intercourse)	1	2	3	4
46. Answer this question only if you have been sexually active: To what extent was sex enjoyable for you?	1	2	3	4

Please go on to the next page

SPECIMEN

**During the past week:**	**Not at All**	**A Little**	**Quite a Bit**	**Very Much**
47. Did you have any pain in your arm or shoulder?	1	2	3	4
48. Did you have a swollen arm or hand?	1	2	3	4
49. Was it difficult to raise your arm or to move it sideways?	1	2	3	4
50. Have you had any pain in the area of your affected breast?	1	2	3	4
51. Was the area of your affected breast swollen?	1	2	3	4
52. Was the area of your affected breast oversensitive?	1	2	3	4
53. Have you had skin problems on or in the area of your affected breast (e.g., itchy, dry, flaky)?	1	2	3	4

SPECIMEN

© Copyright 1994 EORTC Quality of Life Group. All rights reserved. Version 1.0
